# Community‐University Partnerships for Successful Aging Research: The Canadian SuperAging Research Initiative Site

**DOI:** 10.1002/alz70857_104887

**Published:** 2025-12-25

**Authors:** Angela C. Roberts, Elizabeth Finger, Ivan Culum, Emily Narayan, Renaud Jeff, Lindsay Wiley, Desmond O. Oklikah, Joseph B Orange, Bill McIlroy, Karen Van Ooteghem, Andrew Lim, Richard H. Swartz, Robert Bartha, Phyllis Timpo, Amanda Cook Maher, Emily J Rogalski, SuperAging Research Initiative

**Affiliations:** ^1^ Canadian Centre for Activity and Aging, London, ON, Canada; ^2^ Western University, London, ON, Canada; ^3^ Lawson Health Research Institutes, London, ON, Canada; ^4^ University of Western Ontario, London, ON, Canada; ^5^ University of Waterloo, Waterloo, ON, Canada; ^6^ Hurvitz Brain Sciences Research Program, Sunnybrook Research Institute, Toronto, ON, Canada; ^7^ Temerty Faculty of Medicine, University of Toronto, Toronto, ON, Canada; ^8^ Division of Neurology, Department of Medicine, Sunnybrook Health Sciences Centre, Toronto, ON, Canada; ^9^ Sunnybrook Research Institute, Toronto, ON, Canada; ^10^ University of Toronto, Toronto, ON, Canada; ^11^ Centre for Functional and Metabolic Mapping, Robarts Research Institute, London, ON, Canada; ^12^ Robarts Research Institute, University of Western Ontario, London, ON, Canada; ^13^ University of Chicago, Chicago, IL, USA; ^14^ Michigan Alzheimer's Disease Research Center, Ann Arbor, MI, USA; ^15^ University of Michigan, Ann Arbor, MI, USA; ^16^ Healthy Aging & Alzheimer's Research Care (HAARC) Center, University of Chicago, Chicago, IL, USA

## Abstract

**Background:**

SuperAgers—individuals aged 80 and older who perform episodic memory tasks at least as well as those 20 to 30 years younger—provide valuable insights into cognitive resilience and resistance to neurodegeneration. The SuperAging Research Initiative (SRI), led by researchers at the University of Chicago, aims to advance research on SuperAgers, particularly emphasizing increasing Black representation in aging studies. In 2022, the SRI established its first Canadian site at Western University in partnership with the University of Waterloo and Sunnybrook Research Institute, capitalizing on the province's historical significance in Black migration to Canada alongside Western's leadership in aging research. This presentation will outline a strategic university‐community partnership for recruiting and retaining SRI participants in Canada.

**Method:**

In 2023, we commenced recruiting and enrolling a targeted sample of 100 Canadian SuperAgers and controls. Western University is the lead enrollment site. In collaboration with community partners, Western Media/Communications teams, the SRI sites, and SuperAging participants, we developed a multifaceted community‐engaged research (CER) strategy and branding campaign tailored to the Canadian consortium.

**Result:**

We facilitated 16 media appearances and organized five community events. We trained SRI participant volunteers to serve as ambassadors, engaging with organizations, hosting information sessions, and co‐designing marketing materials. Through this campaign, thirteen new strategic partnerships emerged with faith communities, political and community leaders, social clubs, historical societies, medical practices, libraries, and community centers. CER efforts attracted interest from 121 age‐eligible individuals, with 55 completing at least one baseline visit, exceeding our 50% enrollment target, with 0% attrition of eligible participants at two‐year follow‐up. Half of the study enrollments are directly attributed to SRI ambassador activities. Highlighting the persistence required to establish community partnerships, the first Black participant enrolled twenty months after site approvals.

**Conclusion:**

These findings underscore the effectiveness of a multifaceted strategy for recruiting healthy octogenarians and nonagenarians. They also emphasize the importance of involving community partners and peer ambassadors in recruitment and retention efforts. Importantly, our experiences highlight the time and significant resources necessary to cultivate and maintain partnerships and community trust for the successful engagement of individuals 80 and older in research, particularly those from diverse communities.

